# The Influence of Retreated Lithium Slag with a High Content of Alkali, Sulfate and Fluoride on the Composition and the Microstructure of Autoclaved Aerated Concrete

**DOI:** 10.3390/ma17112569

**Published:** 2024-05-27

**Authors:** Dongqing Zhong, Shihong Wei, Hao Zhou, Xiaohang He, Binbin Qian, Bing Ma, Yueyang Hu, Xuehong Ren

**Affiliations:** 1 College of Materials Science and Engineering, Yancheng Institute of Technology, Yancheng 224000, China; zdq811004@ycit.edu.cn (D.Z.); m2872702401@163.com (S.W.); m1733817966@163.com (X.H.); 2 Nanjing Institute of Environmental Sciences, Ministry of Ecology and Environment of the People’s Republic of China, Nanjing 210042, China; zhouhao@nies.org (H.Z.); myyanb@aliyun.com (B.M.); 3 School of Chemistry and Environmental Engineering, Yancheng Teachers University, Yancheng 224002, China; binbinqian-yctu@163.com; 4 State Key Laboratory of Green Building Materials, China Building Materials Academy, Beijing 100024, China

**Keywords:** retreated lithium slag, autoclaved aerated concrete, tobermorite

## Abstract

In this paper, the possibility of retreated lithium slag (RTLS) with a high content of alkali, sulfate and fluoride as a partial replacement for fly ash (FA) to produce autoclaved aerated concrete (AAC) was investigated. The influence of the RTLS dosage on the AAC performance were examined. The composition and microstructure of hydrates as well as the microstructure of the RTLS-FA-based AAC compositions were determined by XRD, FTIR, TG-DSC and SEM. The results illustrated that the incorporation of RTLS changed the crystal structure and the microstructure of the tobermorite. With increased RTLS contents, the morphology of tobermorite was changed, and the grass-like tobermorite gradually transformed into network-like tobermorite. The newly formed tobermorite improved the mechanical performance of the AAC. Compared with the RTLS10, the content of tobermorite in the RTLS30 increased by 8.6%.

## 1. Introduction

**Figure 1:**
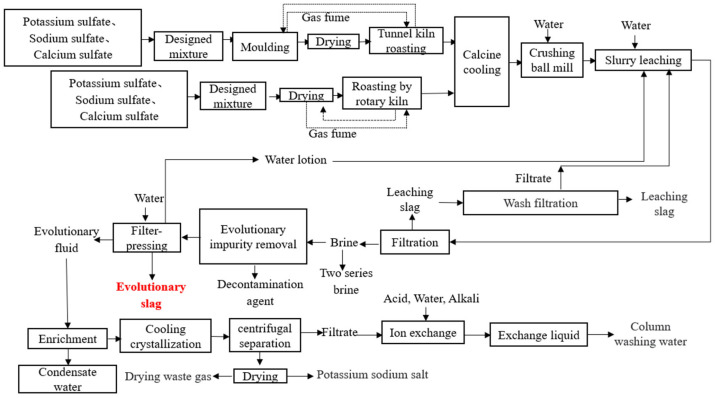
Formation process of the RTLS.

To improve the purification of lithium, lithium slag (LS) has been reused in China, and retreated lithium slag (RTLS) is generated after the brine process. In general, producing one ton of lithium will generate approximately 500 Mt of RTLS. The formation process of RTLS is depicted in Figure 1. To be specific, this process uses potassium sulfate, sodium sulfate and calcium sulfate as raw materials to prepare a lithium-based sample by a mixture design, mold, dry, roast and calcine cool. First of all, water is added into the sample, and then the above mixture is crushed and ball milled, resulting in a slurry. Next, the slurry is leached by water. The leached slag is thus generated as industrial by-products, named LS, in this process. Finally, brine and decontamination agents are added to the slurry to obtain Evolutionary slag (RTLS). As the lithium energy industry develops rapidly, the generation of RTLS has increased sharply. The accumulation of RTLS not only harms human health but also destroys the ecological environment.

It was reported that the composition and proportion of LS are equivalent to those of fly ash (FA) [1,2,3]. Hence, the hydration activities of LS are similar to that of FA. LS has been successfully used as supplementary cementitious materials in China. Compared with the LS, the composition of RTLS was different. The RTLS has higher levels of alkali, sulfate and fluoride than traditional supplementary cementitious material. Based on the composition of RTLS, the utilization of RTLS is limited in the construction industry. Therefore, it is crucial to find a novel approach to utilize the RTLS safely and efficiently.

Autoclaved aerated concrete (AAC) is a green and environmentally friendly porous building material produced by foaming. With its light weight, decent thermal insulation and excellent seismic function, it has attracted attention for a wide range of applications in building materials [4,5]. AAC is mainly produced by siliceous and calcareous materials, the siliceous materials used in AAC containing FA or quartz powder, calcareous materials used in AAC containing lime and OPC. To improve the performance of AAC, the solid wastes were added as raw materials to the preparation of the AAC. Siliceous materials (such as ZSM-5 and iron tailing), calcareous materials (steel slag) and sulfate-containing materials (like phosphogypsum and red gypsum) were successfully utilized in the AAC [6,7,8,9,10,11]. However, the influence of the high-alkali, sulfate and fluoride-containing materials on the composition and the microstructure of the products in AAC has not been investigated. 

In this research, the impact of RTLS on the pH and viscosity of the AAC slurry were investigated. The addition of RTLS with a high content of alkali, sulfate and fluoride on the compressive strength, the evolution of phases, the change in the pore structure and the microstructure of the AAC were also measured.

## 2. Materials and Experimental Methods

### 2.1. Materials

**Figure 2:**
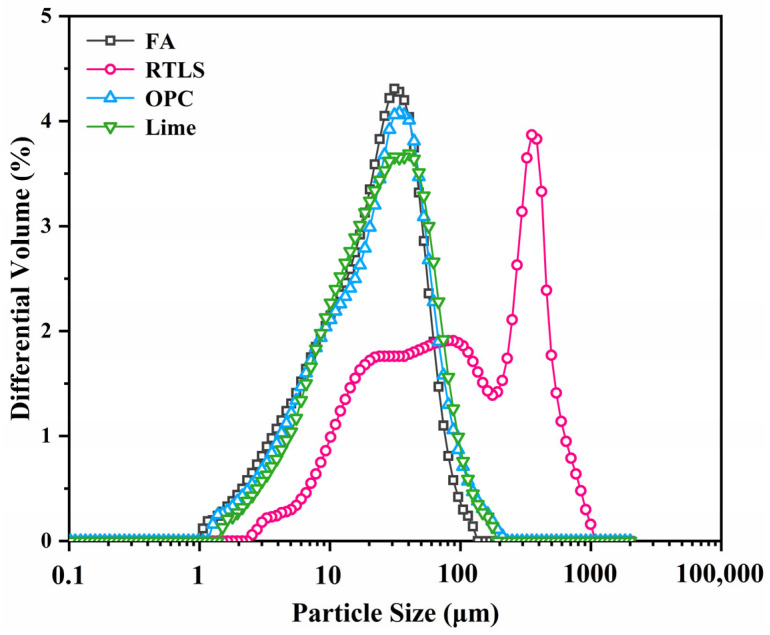
Particle size distributions of the FA, RTLS, OPC and lime.

The RTLS, OPC, lime, FA, Al powder and gypsum were used to prepare the AAC. The RTLS was provided by Jiangxi Ganfeng Lithium Co., Ltd., Xinyu, China. The FA was obtained from Yancheng. The lime was collected from Jiangsu Huanghai Cement Co., Ltd., Nantong, China. The OPC was obtained from Taicang Conch Cement Co., Ltd., Taicang, China. Gypsum and Al powder were supplied by Jiangsu Baling Conch Cement Co., Ltd., Yancheng, China. Elemental compositions and particle size distributions of the RTLS, OPC, FA and lime are shown in Table 1 and Figure 2. The particle sizes of the OPC, FA and lime are smaller than that of the RTLS. The D50 values of the RTLS, FA, OPC and lime are 89.97 µm, 21.07 µm, 23.25 µm and 23.23 µm, respectively.

**Figure 3:**
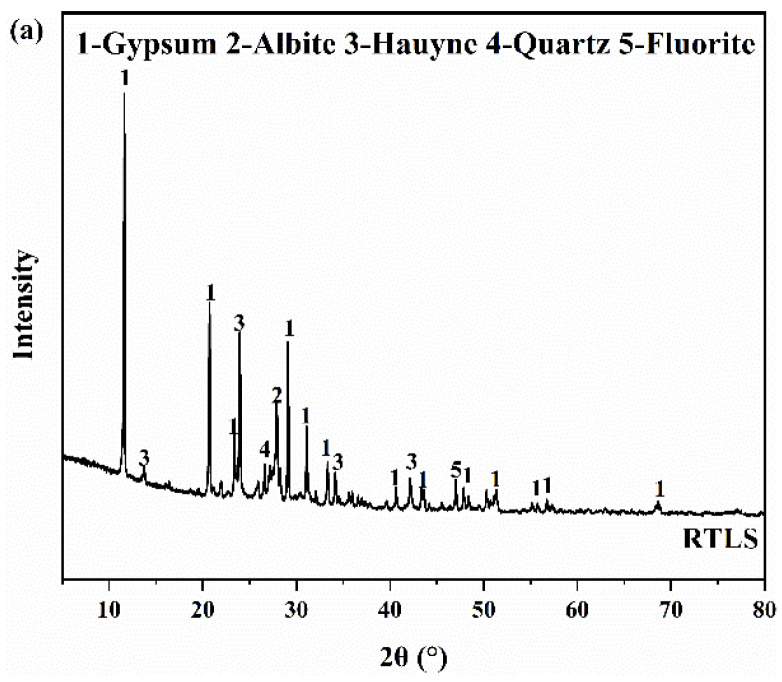

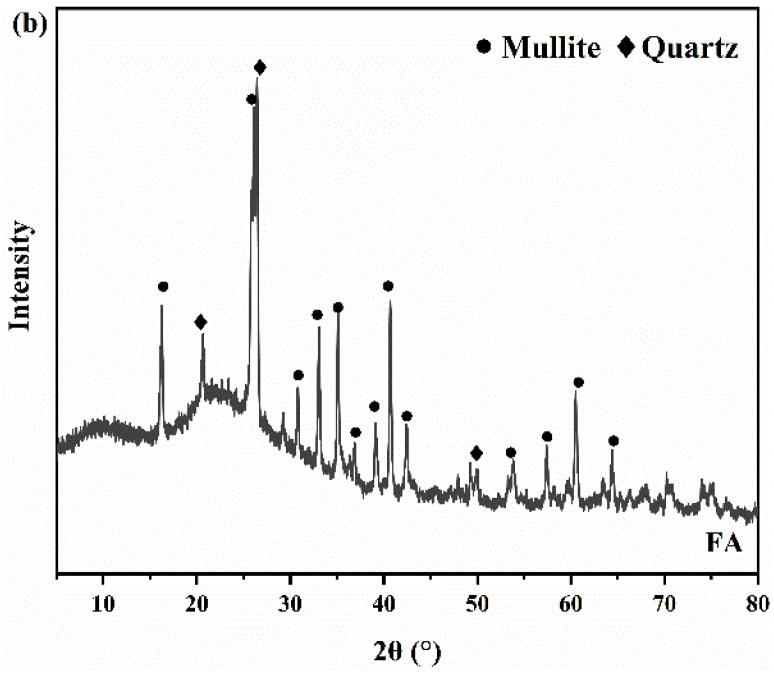
The XRD patterns of the raw materials. (**a**) RTLS; (**b**) FA.

The mineral compositions of the RTLS and FA measured by the XRD are listed in Figure 3. Figure 3a shows that the primary mineral phases of the RTLS are gypsum, albite, hauyne, quartz and fluorite, and the phases of the FA comprise quartz and mullite. The quantitative phase analysis of the RTLS and FA were performed with the X’Pert HighScore Plus software (Version 3.0e). The quantitative analysis result of the RTLS and FA are presented in Table 2 and Table 3.

### 2.2. Sample Preparation

The AAC specimens were produced with RTLS, OPC, FA, lime, Al powder and gypsum as raw materials. The mixing ratios of the AAC samples are shown in Table 4. The FA was replaced by the RTLS in the AAC production at levels of 0%, 10%, 20% and 30%. The water/solid ratio (*w*/*s*) was fixed at 0.7. Samples with different contents of RTLS in the AAC were used to investigate the impact of the RTLS on the properties of the AAC. The AAC was prepared by following these steps. First, the raw materials (RTLS, OPC, lime, gypsum and FA) were mixed for 2 min in a vertical mixer to ensure even mixing. The mixture was then combined with water for stirring for approximately 5 min. Next, the Al powder was added to the paste and blended for another 60 s. The slurry was poured into a mold (100 mm × 100 mm × 100 mm), and the specimens were put into a curing box at 65 °C for 24 h. Eventually, the specimens were prepared at 200 °C with a pressure of 2 MPa for 10 h.

### 2.3. Tests and Methods

The mechanical performance of the AAC was measured using a servo pressure tester (HG-YH200F, Jiangsu Zhuoheng Measurement and Control Technology Co., Ltd., Nanjing, China) in accordance with the requirements of GB/T 11969-2022 [12]. The foaming rate of the paste was determined by the volumetric expansion velocity of the slurry in the measuring cylinder. The viscosity of the slurry was determined in a viscosity measurer (NDG-8S, Shanghai Yueping Science Instrument Co., Ltd., Shanghai, China).

The samples were treated with ethanol for 12 h to stop the hydration. Then, the specimens were dried at 40 °C in a desiccator for 5 h. The dried specimens were ground into powder before the analysis.

The crystalline phases of the raw materials and AAC were analyzed by XRD analysis with CuKα radiation. Specimens were tested over a range of 5°–80° under 40 kV. The scan rate was 5°/min.

For the investigation of the contents of the amorphous and crystalline phases not quantified (ACn) in the raw materials, the external method was used in this study. The external method is generally known as the G-method. The diffractometer constant of the diffractometer operation condition was determined by the α-Al_2_O_3_ (SRM-676a, NIST, Gaithersburg, MD, USA). The crystalline phase purity of the NIST standard reference material SRM-676a was 99.02% ± 1.11%. The content of the phases of the raw materials was calculated by X’Pert HighScore Plus. The scale factors of the phases, the unit cell of the crystalline minerals and the peak shape parameter were refined in this study. The products of the AAC were considered as samples consisting of crystalline phases. The C-S-H gel was reacted to form tobermorite by the autoclaving process.

Thermogravimetric analysis (TG) was used to determine the crystalline phases and amorphous phase. The powder (10 mg) of the AAC was added to an Al_2_O_3_ vessel with N_2_ used as a protection gas. The heating process was from 25 °C to 1000 °C with an increase rate of 10 °C/min.

FTIR analyses of the samples were conducted in a Fourier transform infrared spectrometer (NEXUF-670, Nicolet, Green Bay, WI, USA) with a scanning distance of 4000 and 400 cm^−1^, and the resolution was 4 cm^−1^.

SEM was performed to analyze the microstructures of the samples using a JSM-5610LV. The samples were fractured and fixed to the specimen holder with a gold layer sprayed on the surface to improve the electrical conductivity.

The existence of the porosity and the structure of the pores have an influence on the mechanical properties of the AAC. Mercury intrusion porosimetry (MIP) was used in this study with an Auto Pore IV 9500 apparatus.

## 3. Results and Discussion

### 3.1. The Influence of Alkali, Sulfate and Fluoride on the Performance of Glass in FA with the Coexistence of Lime

The performance of FA used in the OPC is primarily impacted with the chemical and mineral compositions of glass. The mineral composition of glass in FA mainly consists of Al-Si glass in low-CaO FA and Ca-Al-Si glass in high-CaO FA. The content of calcium in glass has a significant influence on the pozzolanic and hydraulic reaction of FA. 

The alumina existing in glass can be react with the calcium hydroxide (Ca(OH)_2_) from lime in AAC. The reaction products may be strätlingite or hydrogarnet (C_3_AH_6_), calcium aluminate hydrate (C_4_AH_13_), ettringite (AFt), calcium monosulfoaluminate and calcium carboaluminate.

With the coexistence of Ca(OH)_2_ and sodium sulfate in concrete, the calcium hydroxide and sodium sulfate can be reacted to gypsum and sodium hydroxide, as shown in the following equation [13]:

(1) $${Ca(OH)}_{2}+{Na}_{2}{SO}_{4}\cdot {10H}_{2}O\to {CaSO}_{4}\cdot {2H}_{2}O+2NaOH+8H_{2}O$$

With the existence of alkali hydroxides, the glass containing the silica and aluminosilica framework is attacked by the OH^−^ and the glass begins to dissolve. As the hydration proceeds, an amorphous gel consisting of alkali and silicate is detected. The change in the gel is affected by the content of calcium and alkali in the solution. With the high content of calcium in the solutions, a C-S-H gel was formed and the alkali-silicate disappeared. With the high content of alkali in the solution, the gels can be N-(A)-S-H and N-S-H. The influence of sulfate on the formation of gels is complicated [14]. Generally, the sulfate is thought to be adsorbed in the C-S-H gel [15,16]. However, some investigations posit that the silicon atoms and aluminum atoms remain [17]. Fluoride mainly delays the hydration of the paste. The alkali in the RTLS reacted with the calcium hydroxide to form potassium hydroxide and sodium hydroxide, as shown in Equation (1). The presence of NaOH and KOH promoted the destruction of the glass structure in the FA.

### 3.2. Viscosity and Foaming Performance of Slurry

The viscosity of the slurry is shown in Table 5. With the increasing content of the RTLS, the viscosity of the slurry initially increased and then decreased sharply. When the content of the RTLS increased to 20 wt%, the slurry showed the highest viscosity. The change in the viscosity can be attributed to the following two factors: (a) the increase in the viscosity results from the activation of the alkali and gypsum to the glass at a small addition of RTLS, and (b) the growing content of the RTLS results in a decrease in cementitious materials in the slurry, which significantly reduces the viscosity of the slurry. It has been shown that viscosity has a significant effect on the foaming rate [18,19].

**Figure 4:**
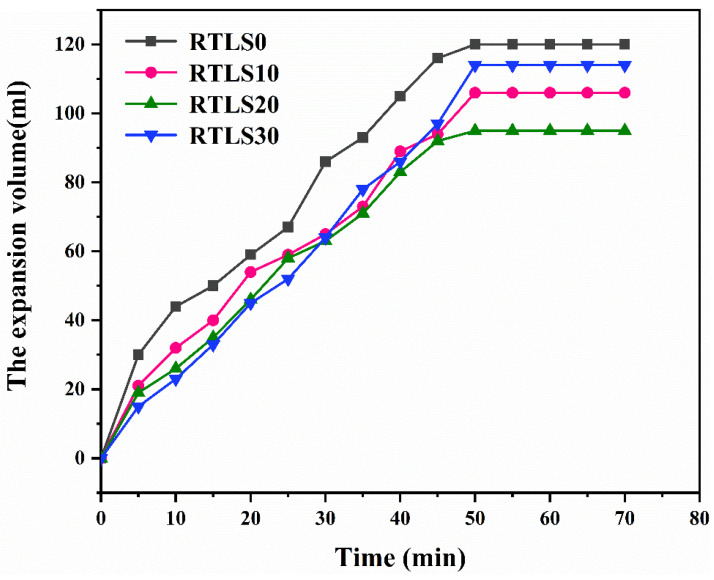
The expansion volume of slurry vs. time.

Figure 4 shows the volume expansion of the slurry. All specimens expand rapidly before 50 min, and all tend to expand smoothly after 50 min. The addition of the RTLS to the slurry has an important effect on the volume expansion. In RTLS0, the glass in the fly ash has been activated by heat and calcium hydroxide [15]. The glass in the FA reacted to form C-S-H gel. With the hydration process, the viscosity of the FA-based slurry increased, and H_2_ bubbles formed quickly [20]. The volume of the slurry increased with time before 50 min. After this, the formation of H_2_ bubbles was limited by the lack of Al powder and the increase in the viscosity. With the addition of RTLS, the glass of the FA was activated by heat, calcium hydroxide, NaOH and gypsum [13]. The main hydrates of the RTLS-FA system were C-S-H gel and AFt. Compared with the RTLS0, the viscosity of RTLS10 and RTLS20 increased significantly with the formation of the C-S-H gel and AFt. The foaming performances of RTLS10 and RTLS20 were limited by the high viscosity. However, in the RTSL30 system, the viscosity of the slurry decreased sharply as a result of the decrease in the cementitious materials. With the high content of alkali and gypsum in RTSL30, the glass in the FA was quickly activated. The viscosity of the slurry increased and H_2_ bubbles quickly formed. Compared to RTSL10 and RTLS20, the expansion volume of RTSL30 achieved the same level at 30 min. After 30 min, the expansion volume of RTSL30 still quickly increased before 50 min.

### 3.3. Compressive Strength

**Figure 5:**
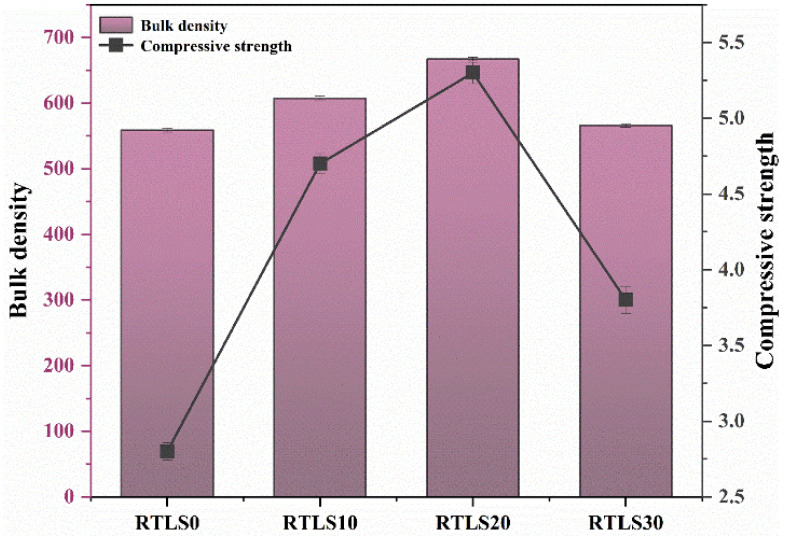
The bulk density and compressive strength of the AAC.

The bulk density and compressive strength of the AAC samples are summarized in Figure 5. The characteristics of the foaming process and the viscosity of the paste have a decisive influence on the bulk density of the AAC [21]. The bulk densities of the RTLS0, RTLS10, RTLS20 and RTLS30 samples were 559, 607, 667 and 566 kg/m^3^, respectively. The addition of the RTLS increased the bulk density of the AAC. The change in the bulk density can be attributed to the following two factors: (a) the increase in the viscosity and (b) the different densities of the raw materials. The results of the bulk density are consistent with those of the expansion volume.

The compressive strengths of the RTLS0, RTLS10, RTLS20 and RTLS30 were 2.8, 4.7, 5.3 and 3.8 MPa, respectively. In the RTLS10 and RTLS20, the increase in the bulk density led to an improvement in the compressive strength. The compressive strength of the AAC was increased to 2.5 MPa when the RTLS content was increased from 10% to 20%. As the content of the RTLS increased to 30%, the bulk density of the RTLS-FA system was similar to the FA system. However, the compressive strength of the AAC was quite different. The difference in the compressive strength can be connected with the formation of anhydrite, which was transformed from gypsum in the RTLS. To sum up, the addition of RTLS can significantly enhance the compressive strength of the AAC, which is in agreement with the alkali-excited cement result [22,23].

### 3.4. XRD Results

**Figure 6:**
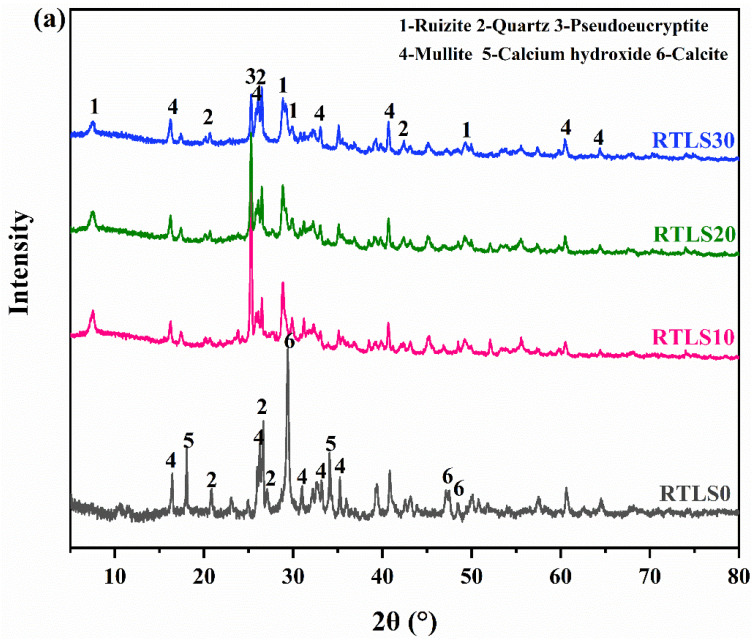

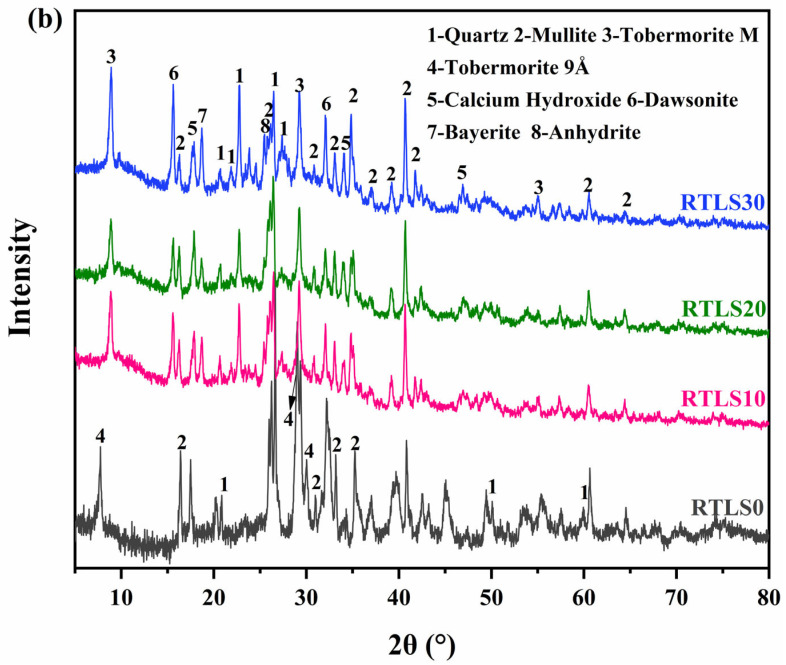
The XRD results of specimens: (**a**) before autoclaving; (**b**) after autoclaving.

Figure 6a,b illustrate the XRD spectra of the RTLS0-RTLS30 samples before autoclaving and after the autoclaving process, respectively. As shown in Figure 6a, the primary crystalline phases of the specimens with the addition of RTLS were quartz, mullite, ruizite and pseudoeucryptite (Li (AlSiO_4_)). The main hydration products in the RTLS0 sample were quartz, mullite, calcium hydroxide and calcite. As the main crystalline phase in the FA, mullites are observed in all samples. Calcite resulting from carbonation was detected in the samples. In addition, it is observed that the diffraction peaks of the pseudoeucryptite become lower as the RTLS contents increase from 10% to 30%.

Figure 6b shows the mineral composition of the AAC. The crystal phases of the FA system are quartz, tobermorite 9Å and mullite. The main crystalline phases are quartz, mullite, tobermorite, calcium hydroxide, anhydrite, dawsonite and bayerite in the RTLS-FA system. The ruizite disappeared in the RTLS-FA system. At the same time, the diffraction peaks for tobermorite (2θ = 8.85°) were formed after autoclaving, which was different from the tobermorite in the FA system. Based on the quantitative results, the two types for tobermorite (comprising 9Å (ICSD:87689) and M (ICSD:40048)) in the RTLS-FA system were formed. Moreover, tobermorite appears in all of the AAC samples, indicating that the formation of tobermorite is not affected by the existence of alkaline, as has been detected in alkali-activated cements and concretes. Anhydrite was also detected in the RTLS-FA system. The compressive strength of the RTLS-FA system may come from the synergistic effect of tobermorite and anhydrite. 

**Figure 7:**
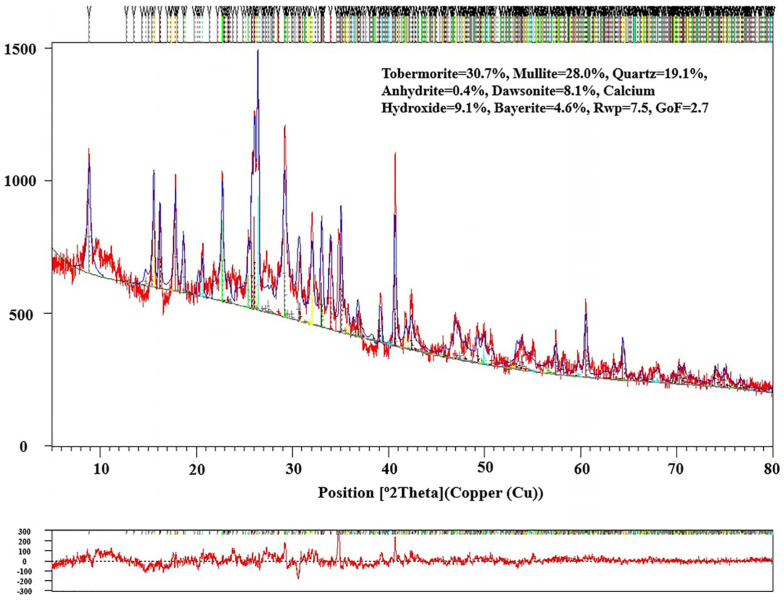
Rietveld refinement of the RTLS10 sample after autoclaving.

The quantitative analysis of the RTLS0-RTLS30 was investigated. The composition of RTLS10 is given in Figure 7. The content of phases in the RTLS0-RTLS30 specimens are shown in Table 6. The addition of RTLS changed the crystal structure of tobermorite. With the content of the RTLS increased in the AAC, the content of tobermorite increased from 30.7% to 39.3%. The increasing content of RTLS can result from the increase in alkali, sulfate and fluoride in the AAC. The high content of alkali, sulfate and fluoride in the system improve the dissolving of the mullite in the autoclaving process. The addition of RTLS results in the residual of calcium hydroxide, which is consistent with the formation of tobermorite in alkali-activated cement and concrete systems.

### 3.5. TG/DSC

**Figure 8:**
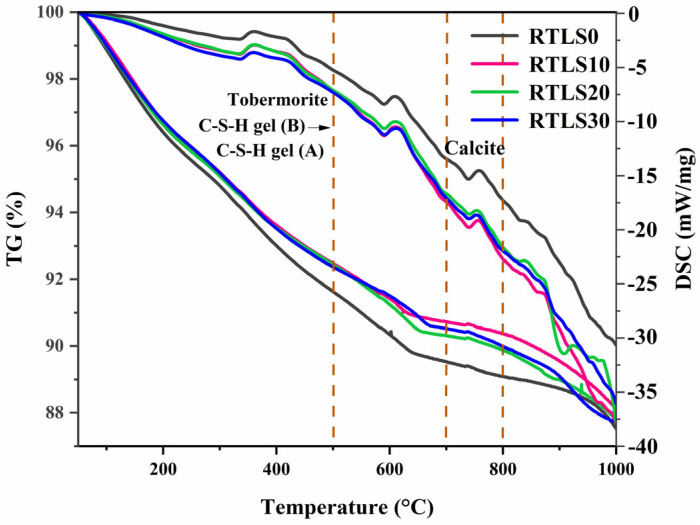
The TG/DSC patterns of the AAC.

The TG/DSC curves of the AAC samples after autoclaving are shown in Figure 8. The crystalline and amorphous phases of the AAC can be reflected in the prominent peaks of the DSC curves. The mass losses of the RTLS0, RTLS10, RTLS20 and RTLS30 are approximately 12.5%, 11.8%, 12.2% and 12.3%, respectively. The RTLS0 has the highest weight loss. The weight loss was roughly divided into four parts according to the temperature. The peak at 300–450 °C is attributed to the dehydration of the tobermorite and the conversion of C-S-H gel (B) to C-S-H gel (A) [24]. The peak of the weight loss appears between 450 °C and 650 °C for two major reasons: (1) The loss of water in the hydration products. (2) The release of CO_2_ also generates the loss of mass [25]. With further heating, the endothermic peaks from 700 °C to 800 °C, which are attributed to the decomposition of calcite, are consistent for the XRD results [26]. The final phase of weight change existed from 800 °C to 1000 °C, and the exothermic peaks at approximately 880 °C are ascribed to the transformation of the C-S-H gel (B) into wollastonite in the RTLS20 sample.

### 3.6. FTIR

**Figure 9:**
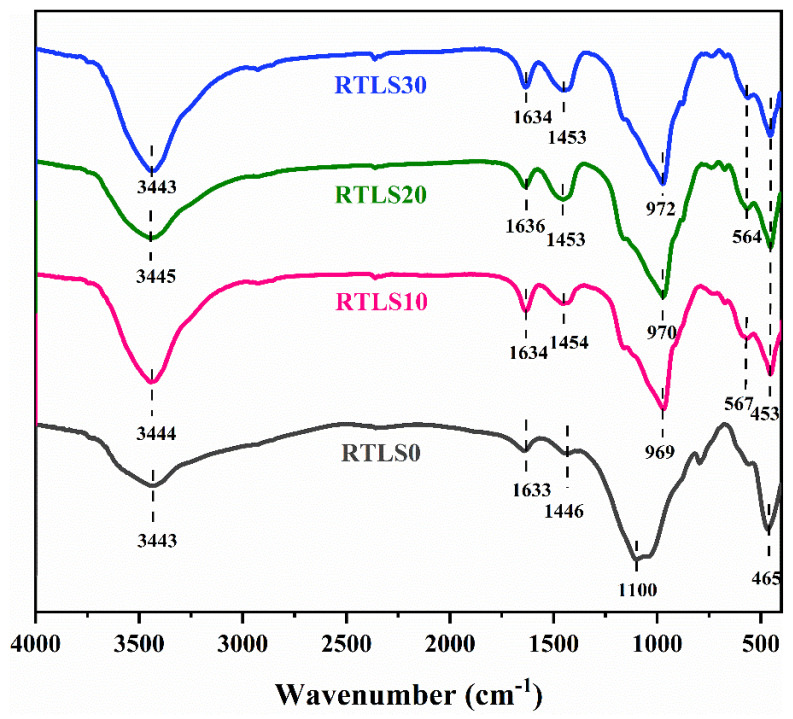
FTIR patterns of the AAC.

The FT-IR spectrum of the AAC specimens over the wavenumber range between 4000 and 400 cm^−1^ are given in Figure 9.

The bands at about 3343 cm^−1^ belong to the bending vibrational modes of water, the C-S-H gel, portlandite and tobermorite [27]. The bands at approximately 1636–1633 cm^−1^ were considered to be symmetrical stretching for the O-H in water [18]. The bands observed at approximately 1454 cm^−1^, 1453 cm^−1^ and 1446 cm^−1^ correspond to the vibrational patterns of the CO_3_^2−^ in the reaction products, also detected by the XRD [28]. The 1100 cm^−1^ band corresponds to the Si-O-T (where T can be Al or Si) vibrations of the FA-based AAC. However, the Si-O-T band moved to 970 cm^−1^ from 1100 cm^−1^. A lower wavenumber for the Si-O-T bands is considered a decreased polymerization degree of the C-S-H gel. However, with the incorporation of Al in the structure of the C-S-H gel in the RTLS-FA system, the wavenumbers for the Si-O-T bands were shifted to lower wavenumbers. This result is consistent with the formed of C-(N)A-S-H gels in alkali-activated slag and metakaolin geopolymers [29]. The bands at 465 cm^−1^ and 453 cm^−1^ correspond to the symmetrical oscillations of Al-O-Si or Si-O-Si bonds [30]. The existence of these bands is considered to represent C-A-S-H-like phases in the system.

### 3.7. SEM and EDS Analysis

To analyze the effect of the RTLS on the microstructure of the samples after autoclaving, SEM and EDS analyses are used in this investigation.

**Figure 10:**
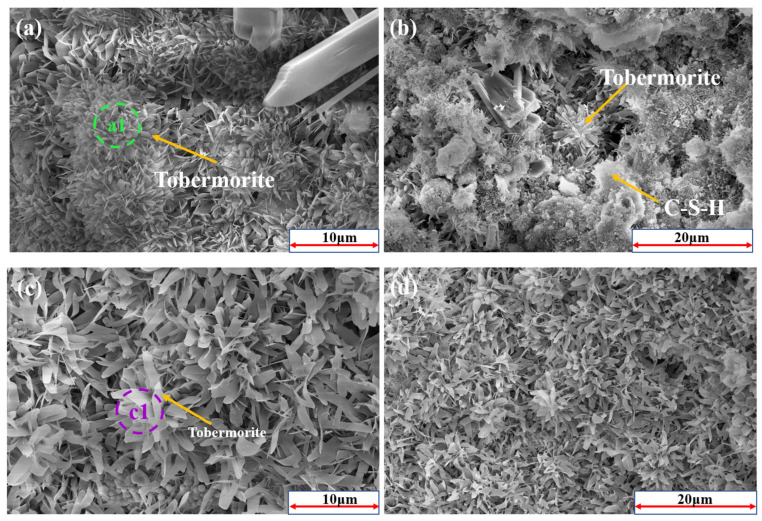

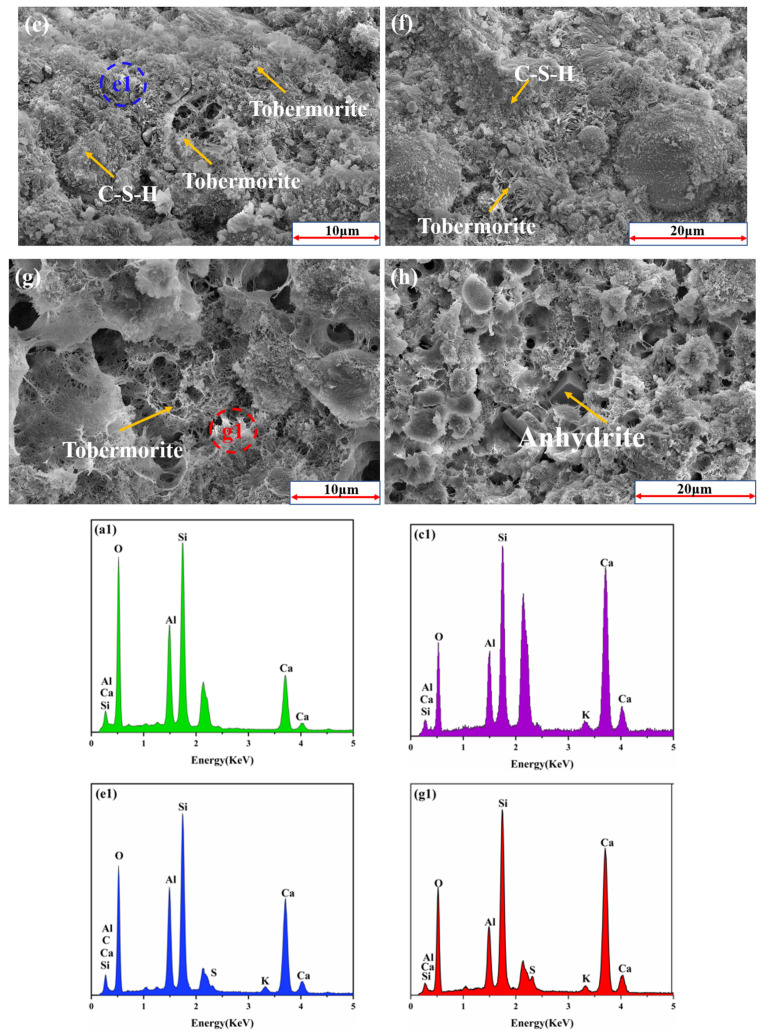
The SEM patterns of the AAC samples: (**a**,**b**,**a1**) RTLS0; (**c**,**d**,**c1**) RTLS10; (**e**,**f**,**e1**) RTLS20; (**g**,**h**,**g1**) RTLS30.

The SEM figures of the fly ash-based system after autoclaving are presented in Figure 10. The major hydrates were grass-like tobermorite and C-S-H gel. Grass-shaped grains have a lower Ca/Si ratio than needle-like tobermorite grains (Ca/Si > 1), which is consistent with the EDS results (Figure 10(a1)) [7].

Figure 10(c–h) are SEM images of the specimens with the addition of RTLS after autoclaving. The major mineral composition of the AAC produced in the autoclaving process was tobermorite, C-S-H gels and anhydrite. The microstructure of the RTLS–fly ash system changed from grass-like tobermorite to network-like tobermorite.

In the RTLS10, the addition of RTLS improved the formation and crystal growth of tobermorite. Compared to the fly ash-based AAC (RTLS0), a denser and better crystalline tobermorite was detected in the RTLS10 (Figure 10c,d). However, the microstructure of the RTLS–fly ash system evidently changed as the RTLS content increased. The grass-like tobermorite in the RTLS10 (Figure 10(c1)) changed to network-like tobermorite in the RTLS20 (Figure 9e,f and Figure 10(e1)) and RTLS30 (Figure 10g,h and Figure 10(g1)). At the same time, anhydrite interlacing with tobermorite and the C-S-H gel is present in the RTLS–fly ash system, which makes the pore structure of the specimen considerably more compact and contributes to the strength. The results of the SEM/EDS analysis are consistent with those of the XRD, TG/DSC and FTIR analyses.

### 3.8. MIP Analysis of the Samples

The pore structure of the AAC was determined via MIP. As a porous material, the size of the pores in the AAC range from the millimeter scale to the nanoscale. The pore size and porosity have a dramatic effect on the AAC performance [31].

The porosity of the AAC specimens is presented in Table 7. The porosity values observed for the RTLS0, RTLS10, RTLS20 and RTLS30 were 61.76%, 62.97%, 62.3% and 62.15%, respectively. With the addition of the retreated lithium slag, the porosity gradually increased compared to the RTLS0. Porosity associated with foaming, which is higher for higher porosity, results in less satisfactory foaming, a denser structure in the AAC and a stronger compressive strength [32].

**Figure 11:**
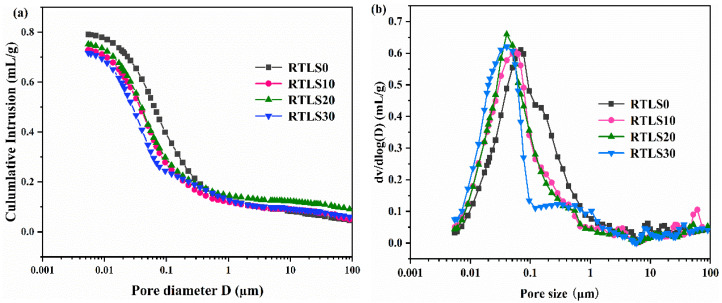
Intruded Hg volume vs. pore diameter for the AAC: (**a**) cumulative plot; (**b**) derivative plot.

The pore structure of the AAC specimens, characterized by MIP, is presented in Figure 11. Figure 11a demonstrates the cumulative pore volume of the sample RTLS0–RTLS30. It is observed that the cumulative intrusion invasion of mercury decreased with the increasing dosage of the retreated lithium slag in the range of pore sizes from 0.01 to 0.1 µm. The pore diameter distribution of the specimen is presented in Figure 11b. Compared with the RTLS0, the pore size of the RTLS–fly ash system (RTLS10, RTLS20 and RTLS30) specimens between 0.01 and 0.1 µm is shifted to the left. The change in the pore structure is considered to enhance the strength.

**Figure 12:**
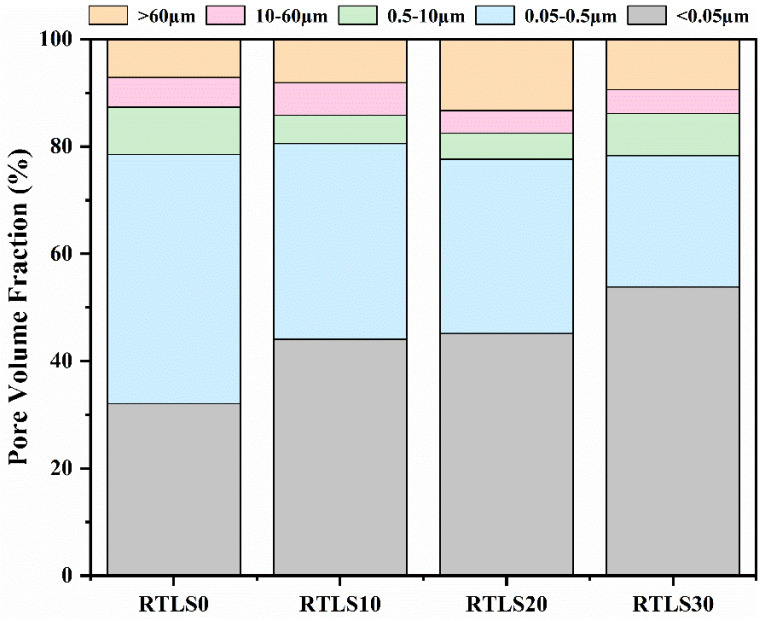
Pore size distribution of the autoclaved samples.

The properties and densities of the samples are related to the pore size distribution [33,34]. According to the different sizes of the pores, they can be divided into the following three types: gel pores (<0.05 µm), capillary interstice pores (0.05–60 µm) and macropores (>60 µm). Figure 12 shows the pore size distributions of the autoclaved samples. As compared to the RTLS0, with the increase in the RTLS content, the amount of gel pores significantly increases in the samples. Clearly, the inclusion of RTLS facilitates the further refinement of the pores in the samples. As can be seen from the figure, the pores were composed mainly of gel pores and capillary pores in the AAC specimens, allowing them to form a denser structure with tobermorite, C-S-H gel and anhydrite, resulting in a facilitated development in the strength in the samples.

## 4. Discussion

**Figure 13:**
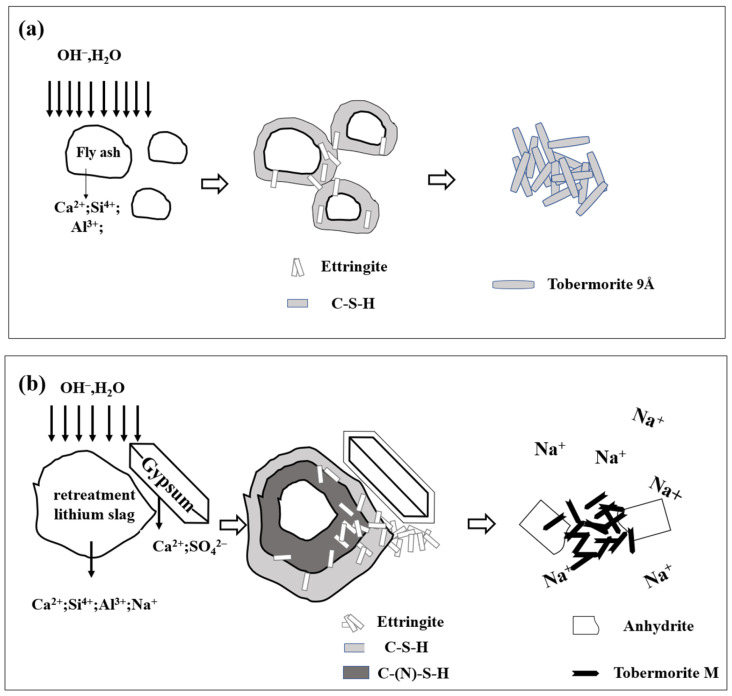
The reaction mechanism for the AAC: (**a**) FA-based; (**b**) RTLS-FA system.

The hydration mechanism of the fly ash-based AAC is shown in Figure 13a. The existence of Ca^2+^ dissolved from lime and cement in the solution reacts with the Al-Si glass in fly ash to form the C-S-H gel. The transformation of the C-S-H gel into tobermorite in the autoclaving process is shown according to Equation (2).

The reaction mechanism of the RTLS-FA system is exhibited in Figure 11b. Due to the higher alkali and sulfur content in the RTLS, the glass in the FA was activated in the following three ways: (i) alkaline activation; (ii) lime activation; (iii) sulfate activation.

With the addition of the RTLS to the slurry, the alkali, sulfate and Ca^2+^ were dissolved in the solution. The glass in the fly ash was activated by those ions. The C-(N)-S-H gel, C-S-H gel and AFt were formed as hydrates before autoclaving, as shown in Equations (3) and (4), typically observed in sulfate-GGBFS geopolymers [35]. With the autoclaving process, the C-(N)-S-H gel and C-S-H gel were changed to tobermorite, as shown in Equation (5). The formation of tobermorite in the RTLS-FA system is different from that in the FA-based AAC. The results were proven by the XRD and FTIR analyses. Crystalline phases (tobermorite and sodium-zeolite) were detected in the alkali-activated slag subjected to hydrothermal treatment.

(2) $${Ca}^{2+}+Al-Si\;glass\overset{hydration}{\to }C-S-H\;gel\overset{hydrothermal\;synthesis}{\to }C_{5}S_{6}H_{5}$$

(3) $${Na}^{+}+{Ca}^{2+}+Al-Si\;glass\overset{hydration}{\to }C-\left(N\right)-S-H\;gel$$

(4) $${Ca}^{2+}+{SO}_{4}^{2-}+Al-Si\;glass\overset{hydration}{\to }AFt$$

(5) $$C-\left(N\right)-S-H\;gel\overset{hydrothermal\;synthesis}{\to }{Na}^{+}+C_{5}S_{6}H_{5}$$

(6) $$AFt\overset{hydrothermal\;synthesis}{\to }\;Ca{SO}_{4}$$

The AFt was decomposed to anhydrite as in Equation (6), typically observed in the phosphogypsum- and red gypsum-based AAC. At the same time, the crystalline C-S-H gel was formed in the RTLS-FA system. The addition of RTLS to the slurry changed the mineral composition of the AAC. However, the compressive strength of the AAC was improved by the addition of the RTLS due to the synergistic effect of tobermorite and anhydrite.

## 5. Conclusions

This paper has proposed a novel AAC preparation method from the partial replacement of fly ash with retreated lithium slag, and the influences of retreated lithium slag on the compressive strength, hydration products, foaming process and bulk density of the AAC were examined. From the current research, the main conclusions are drawn as follows:

1. The addition of RTLS can change the mineral composition of the AAC. The formation of tobermorite in the RTLS-FA system is different from that in the FA system.
2. The addition of RTLS changed the microstructure of the tobermorite. As the content of the RTLS increased, the microstructure changed from grass-like tobermorite to network-like tobermorite.
3. The addition of RTLS can improve the AAC performance, which is attributed to the synergistic effect of tobermorite and anhydrite. Compared with the RTLS0, the compressive strength of the RTLS30 was enhanced by 10 MPa.

## Figures and Tables

**Table 1:** Chemical compositions of the RTLS, FA, lime and OPC (wt%).

Component	SiO_2_	Al_2_O_3_	CaO	MgO	Fe_2_O_3_	SO_3_	Na_2_O	K_2_O	TiO_2_	Loss of Ignition
RTLS	32.44	19.09	12.21	0.62	2.67	12.48	5.05	6.45	0.07	5.89
FA	46.21	27.23	9.52	4.73	3.56	0.39	0.51	0.70	-	5.60
Lime	1.22	1.04	95.5	-	1.23	-	-	-	-	1.01
OPC	22.26	5.89	60.17	2.35	2.96	2.49	0.29	0.71	-	2.88

**Table 2:** The phase composition of the RTLS.

Phase (RTLS)	Phase Composition (g/100g)
Gypsum (ICSD: 27,221)	44.04
Quartz (ICSD: 174)	2.02
Hauyne (ICSD: 24,441)	12.82
Albite (ICSD: 9287)	38.80
Fluorite (ICSD: 28,730)	2.32
Total	100

**Table 3:** Phase component of FA.

Phase (FA)	Phase Composition (g/100g)
Mullite (ICSD: 89,721)	25.45
Quartz (ICSD: 174)	35.76
Amorphous	38.79
Total	100

**Table 4:** Mix proportions of each sample (wt%).

Sample	RTLS	OPC	FA	Lime	Al Powder	Gypsum	Ca/Si
RTLS0	0%	17%	70%	10%	0.225%	3%	0.78
RTLS10	10%	17%	60%	10%	0.225%	3%	0.82
RTLS20	20%	17%	50%	10%	0.225%	3%	0.85
RTLS30	30%	17%	40%	10%	0.225%	3%	0.90

**Table 5:** The viscosity of the samples.

Samples	RTLS0	RTLS10	RTLS20	RTLS30
Viscosity (mPa·s)	1120	1254	1380	1144

**Table 6:** The phase composition of the RTLS0-RTLS30 samples.

Phase	RTLS0	RTLS10	RTLS20	RTLS30
Tobermorite 9Å (ICSD: 87,689)	46.8%	-	-	-
Tobermorite M (ICSD: 40,048)	-	30.7%	32.2%	39.3%
Mullite (ICSD: 89,721)	25.5%	28.0%	26.5%	18.0%
Quartz (ICSD: 89,280)	27.7%	19.1%	15.1%	18.2%
Anhydrite (ICSD: 1956)	-	0.4%	4.9%	1.6%
Dawsonite (ICSD: 17,000)	-	8.1%	11.0%	13.0%
Calcium Hydroxide (ICSD: 15,471)	-	9.1%	7.2%	4.4%
Bayerite (ICSD: 26,830)	-	4.6%	3.1%	5.5%
Total	100%	100%	100%	100%

**Table 7:** The porosity of the AAC samples.

Specimen No.	Porosity (%)
RTLS0	61.76
RTLS10	62.97
RTLS20	62.30
RTLS30	62.15

## Data Availability

Data will be made available on request. The data are not publicly available due to information that could compromise reseach participant privacy.
